# Nationwide analysis of the relationships between mental health, body mass index and tinnitus in premenopausal female adults in Korea: 2010–2012 KNHANES

**DOI:** 10.1038/s41598-018-25576-5

**Published:** 2018-05-04

**Authors:** Dong-Hee Lee, Young Soo Kim, Hiun Suk Chae, Kyungdo Han

**Affiliations:** 1 Epidemiology Study Cluster of Uijeongbu St. Mary’s Hospital, Uijeongbu St. Mary’s Hospital, College of Medicine, The Catholic University of Korea, Uijeongbu, Korea; 2 Department of Otolaryngology-HNS, College of Medicine, The Catholic University of Korea, Seoul, Korea; 3 Department of Internal medicine, College of Medicine, The Catholic University of Korea, Seoul, Korea; 4 Department of Biostatistics, College of Medicine, The Catholic University of Korea, Seoul, Korea

## Abstract

Tinnitus is related to serious comorbidities such as suicidal ideation and attempts. Body mass index (BMI) is associated with auditory symptoms including hearing loss. The aim of this nationwide, population-based, cross-sectional study was to evaluate the relationship between mental health, body mass index and tinnitus in a Korean premenopausal female population. This study analyzed data from the Korea National Health and Nutrition Examination Surveys in 2010–2012. Data were collected from 4628 19 years or older, premenopausal women. After adjustments, underweight premenopausal women exhibited a higher odds ratio for tinnitus (odd ratio = 1.54; 95% confidence interval = 1.14–2.08) compared with women of normal weight. Moderate and severe tinnitus was highly prevalent in underweight as well as extremely obese women. The prevalence of perceived stress, melancholy, and suicide ideation was significantly higher in women with tinnitus. The prevalence of perceived stress and suicide ideation was significantly higher in underweight women with tinnitus, but that of melancholy was significantly lower. This study demonstrated that underweight premenopausal Korean women had a higher risk of tinnitus, which has grown in importance as a public health issue. Women with tinnitus experience perceived stress and suicide ideation more frequently, but melancholy less frequently than women without.

## Introduction

Tinnitus is one of common problems that can have considerable negative effects on the quality of life^1,2^. Tinnitus is generally defined as an individual’s perception of sound in the absence of external auditory stimulus. Its prevalence has been shown to be 4.6–30% in several population-based studies in different countries^1,2^, although the prevalence of troublesome tinnitus is lower. Park *et al*.^3^ analyzed data from the 2009–2011 Korean National Health and Nutrition Examination Surveys (KNHANES) and demonstrated that tinnitus was more prevalent in Korean women than men.

Although body mass index (BMI) is associated with reduced hearing, and tinnitus is closely related to hearing loss, limited data are available on the association between tinnitus and BMI^4–8^. Underweight individuals may have increased risk of morbidity and mortality, but cultural pressure leads many women to desire a slimmer body shape and occasionally adopt excessive weight-control efforts to maintain a thin body, especially young women. Because self-attitude about one’s body shape is associated with cultural canons rather than objective parameters, self-satisfaction with body shape or image is modulated subjectively and determined by personality traits, self-esteem, and locus of control. Negative self-attitude about body weight is common, especially in young women. The fashion of slenderness is a rising issue. Many women tend to consider their body weight as a determinant of their body shape or image and use various slimming diets, despite a lack of objective reasons.

Since negative self-attitude about body weight is a principal determinant of female body image, we hypothesized that underweight or obesity might be a major factor associated with prevalence or severity of tinnitus in women. Considering that hormonal changes during pregnancy, menopause, or following hormone replacement therapy may influence tinnitus^9^, this study focused on premenopausal women. This study investigated the relationship between tinnitus and BMI in premenopausal women using nationwide, population-based, cross-sectional data from South Korea. Secondary aim of this study was to evaluate the association between mental health status and BMI in in premenopausal women with tinnitus.

## Methods

### Study design and participants

This study used data collected by KNHANES in 2010–2012. Since 1998, the KNHANES has been conducted regularly all over the nation as a cross-sectional, population-based health examination and survey by the Division of Chronic Disease Surveillance, Korea Centers for Disease Control and Prevention, Ministry of Health and Welfare in South Korea. The survey includes complex, stratified, multistage sample design feature of probability-cluster, that was designed according to geographic area, sex, and age group was used to select household units. Therefore, its sample represents the total noninstitutionalized civilian population of South Korea.

**Figure 1:**
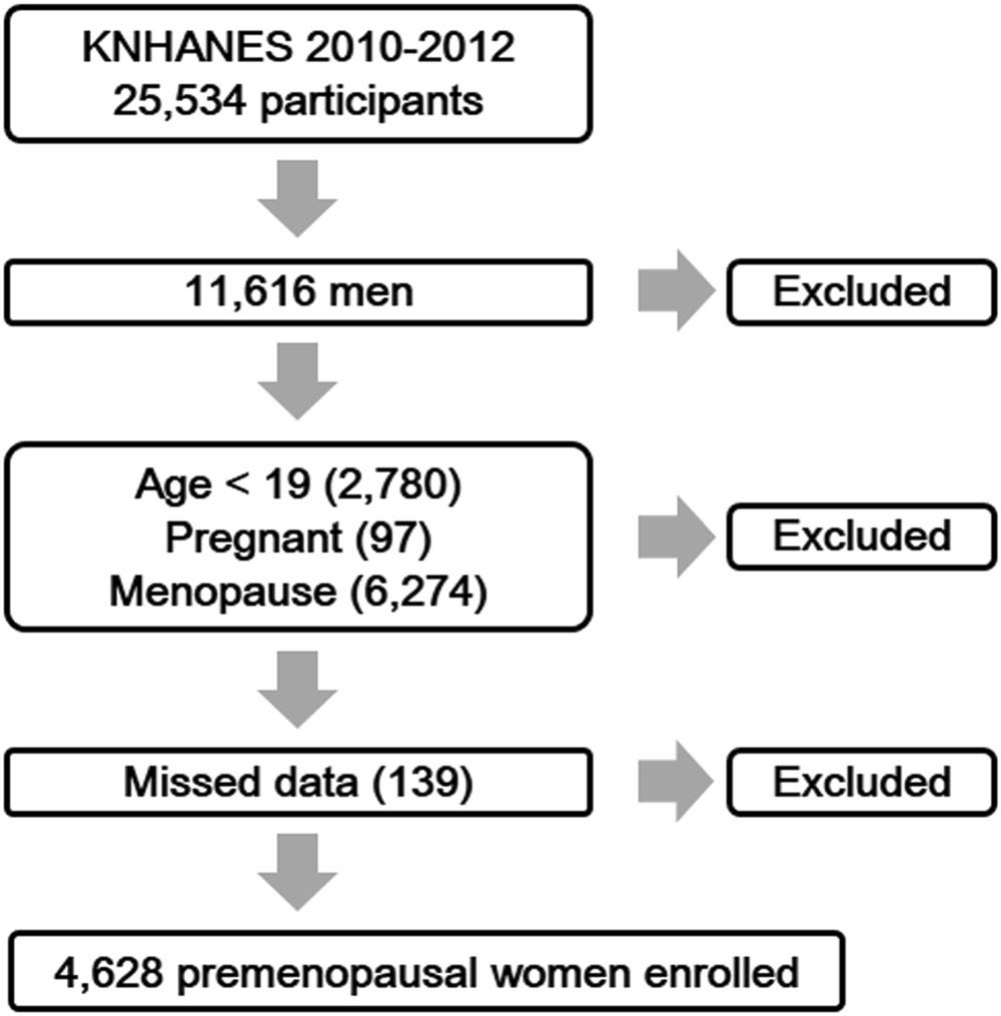
Flow chart of participant enrollment.

A total of 25,534 individuals participated in KNHANES in 2010–2012. Among the women (n = 13,918), we excluded from analysis those younger than 19 years (n = 2,780), pregnant (n = 97), postmenopausal with artificial menopause (hysterectomy or bilateral oophorectomy) (n = 6,274), and those whose records had missing values (n = 139). Thus, 4,628 premenopausal female adults were included in analysis (Fig. 1).

### Anthropometric, health-related, and lifestyle factors

BMI (kg/m^2^) was calculated by dividing body weight (kg) by square of height (m). We used the definitions of the Western Pacific Region of the World Health Organization^10^: underweight BMI < 18.5 kg/m^2^, normal weight 18.5 ≤ BMI < 23 kg/m^2^, overweight 23 ≤ BMI < 25 kg/m^2^, obesity 25 ≤ BMI < 30 kg/m^2^, and extreme obesity BMI ≥ 30 kg/m^2^.

Smoking status was classified as current or ever smoker. Alcohol intake status was classified as ≥2 drinks per week, ≤1 drink per week to ≥2 drinks per month, ≤1 drink per month or non-drinker. High-risk alcohol intake was defined as alcohol intake ≥2 drinks per week and 7 or more standard-sized drinks per session (5 standard-sized drinks for women).

High-intensity physical activity was defined as hard exercise such as jogging, climbing, or fast cycling. Moderately intense physical activity was defined as moderate exercise such as slow swimming, doubles tennis, or table tennis. High-intensity physical activity for ≥20 min per episode and ≥3 days per week or moderate-intensity physical activity for ≥30 min per episode and ≥5 days per week or walking for ≥30 min per episode and ≥5 days per week were defined as physical activity.

Education level was classified as high if the respondent completed college/university or above. Household income was stratified into three tertiles of low, middle, and high. Residential area was categorized into urban or rural, with urban areas including both large and small cities. Types of living arrangements were grouped into living alone, living with a spouse, living with offspring, and living with other family members.

In this study, chronic diseases were diabetes mellitus, hypertension, hypercholesterolemia, hypertriglyceridemia, hypo-high density lipoprotein cholesterolemia, chronic renal disease, and metabolic syndrome.

### Definitions of menopause and tinnitus

Menopause was defined as the absence of a menstrual period for 12 months following the final menstrual period. This study defined postmenopausal status as self-reported cessation of menstruation for more than 1 year and excluded women who had undergone hysterectomy or bilateral salpingo-oophorectomy.

The tinnitus-related question in KNHANES was “Did you ever hear a sound (buzzing, hissing, ringing, humming, roaring, machinery noise) originating in your ear within the past year?” If the answer was “yes,” the participant was required to answer the next question “Does your tinnitus annoy you?” and mark one of the following: (1) My tinnitus is not annoying; (2) My tinnitus is annoying and makes me nervous; or (3) My tinnitus is so annoying that I can hardly sleep at night. Degree of tinnitus had four categories: controls, mild tinnitus, moderate tinnitus, and severe tinnitus. Participants who answered “no” to the tinnitus-related question were classified as controls. For the second question, those who marked “My tinnitus is not annoying” were classified as mild, those who marked “My tinnitus is annoying and makes me nervous” were classified as moderate, and those who marked “My tinnitus is so annoying that I can hardly sleep at night” were classified as severe tinnitus.

### Mental health survey

Three mental health dimensions of perceived stress, melancholy, and suicidal ideation were included in the health status and mental health domains of KNHANES. To assess the level of perceived stress, participants answered ‘none’, ‘mild’, ‘moderate’, or ‘severe’ to a question “How stressed are you on a daily basis?” If participants reported moderate to severe, they were considered as having stress. Melancholy was screened using the Korean version of the World Health Organization’s Composite International Diagnostic Interview-Short Form (CIDI-SF) and participants who answered “yes” to the question “Have you ever felt sad or desperate for two consecutive weeks during the past year so that your daily life is hindered?” were considered as having melancholy. To assess suicide ideation, participants answered to the question “Have you ever thought about committing suicide in the last 12 months?” If the participants answered “yes”, they were considered as having suicide ideation.

### Ethical approval and informed consent

After approval by the Institutional Review Board (IRB) of the Korea Centers for Disease Control and Prevention, all KNHANES surveys were conducted with participants’ informed consent. This study was also approved by the IRB (policy number: UC16EISI0127), which waived the need for informed consent, because this retrospective analysis included the dataset of national surveillance and did not contain personally identifiable information.

### Statistical analysis

For statistical analyses, we used SAS software (version 9.2; SAS Institute, Cary, NC) and SUDAAN software (release 10.1; Research Triangle Institute, Research Triangle Park, NC). In this study, we used sample weights in all analyses to produce estimates that represented the noninstitutionalized civilian Korean population.

The epidemiologic characteristics of participants were expressed as mean ± standard error (SE) or number and prevalence (SE), as appropriate, for all participants. Sample weights were used to obtain SEs for prevalence. We used Student’s *t*-test or chi-square test (using the SURVEYMEANS or SURVEYFREQ procedures in SAS, respectively, to reflect sample weights) to evaluate differences in the anthropometric, health-related, and lifestyle characteristics of women with tinnitus vs. women without tinnitus.

Multivariate adjusted logistic regression analysis was performed to calculate odds ratios (ORs) and 95% confidence interval (CIs) for associations between tinnitus and BMI. We used the SURVEYLOGISTIC procedure in SAS for logistic regression analyses.

All reported P values were 2-tailed, and a P < 0.05 was considered statistically significant.

## Results

### Basic characteristics of the study population

Anthropometric, health-related, and lifestyle factors for premenopausal women with and without tinnitus are presented in Table 1. Women with tinnitus showed a higher prevalence of ever smoking and lowest quartile income than women without tinnitus. In women with tinnitus, proportions living with a spouse and with a higher educational level were significantly lower. The proportion of women with parity was lower, but the proportion of women with irregular menstruation was higher than women without tinnitus. The mean age of women with tinnitus was significantly lower than that of women without tinnitus.

**Table 1:** Summary of anthropometric, health-related, and lifestyle factors in the study population. Data are presented as mean ± standard error (SE) or %.

	Tinnitus		
	No	Yes	P
	N = 3696	N = 932	
Age, years	35.8 ± 0.2	34.0 ± 0.4	<0.001
BMI, kg/m^2^	22.6 ± 0.1	22.5 ± 0.2	0.593
<18.5	N = 327	N = 104	
18.5≤ and <23	N = 1974	N = 471	
23≤ and <25	N = 629	N = 153	
25≤ and <30	N = 621	N = 162	
30≤	N = 145	N = 42	
Waist circumference, cm	75.0 ± 0.2	74.7 ± 0.4	0.546
Duration of sleep, hours	7.04 ± 0.02	6.98 ± 0.05	0.290
Ever smoker (yes), %	9.1 (0.6)	13.0 (1.4)	0.003
High-risk drinker (yes), %	3.3 (0.4)	2.9 (0.7)	0.594
Physical activity (yes), %	16.7 (0.8)	17.9 (1.7)	0.483
Residential area (urban), %	85.8 (1.5)	87.5 (1.8)	0.281
Spouse (yes), %	82.6 (1.3)	78.1 (2.1)	0.029
Occupation (yes), %	57.9 (1.0)	55.3 (2.1)	0.258
Education (college/university or above), %	43.7 (1.1)	38.2 (2.0)	0.011
Income (lowest quartile), %	7.7 (0.7)	12.7 (1.5)	<0.001
Noncontraceptive female hormone use (yes), %	1.9 (0.3)	2.3 (0.6)	0.520
Parity (yes), %	67.8 (1.1)	58.2 (2.1)	<0.001
Irregular menstruation (yes), %	14.2 (0.7)	19.7 (1.7)	0.001

### Mental health, BMI and Tinnitus in premenopausal women

After adjusting for confounding factors, the estimated, weighted crude prevalence of perceived stress, melancholy, and suicide ideation was calculated according to tinnitus and BMI, based on the results of the univariate analysis (Supplementary Table S1A and B). The prevalence of perceived stress, melancholy, and suicide ideation was significantly higher in women with tinnitus (P < 0.001). Differences in the prevalence of perceived stress (P = 0.007) and suicide ideation were significant (P < 0.001) and were the highest in the group with extreme obesity (≥30 kg/m^2^), followed by the group that was underweight (<18.5 kg/m^2^). Prevalence of melancholy was highest in the extreme obesity group, but differences were not significant. The prevalence of perceived stress and suicide ideation was significantly higher in underweight women with tinnitus, but the prevalence of melancholy was significantly lower (P < 0.001) (Supplementary Table S1C). Univariate analysis showed that the prevalence of perceived stress was significantly highest in underweight women with tinnitus. Our study showed that the prevalence of melancholy and suicide ideation was significantly highest in women with tinnitus who were not underweight. These findings may reflect the relationship between mental health status and BMI.

**Figure 2:**
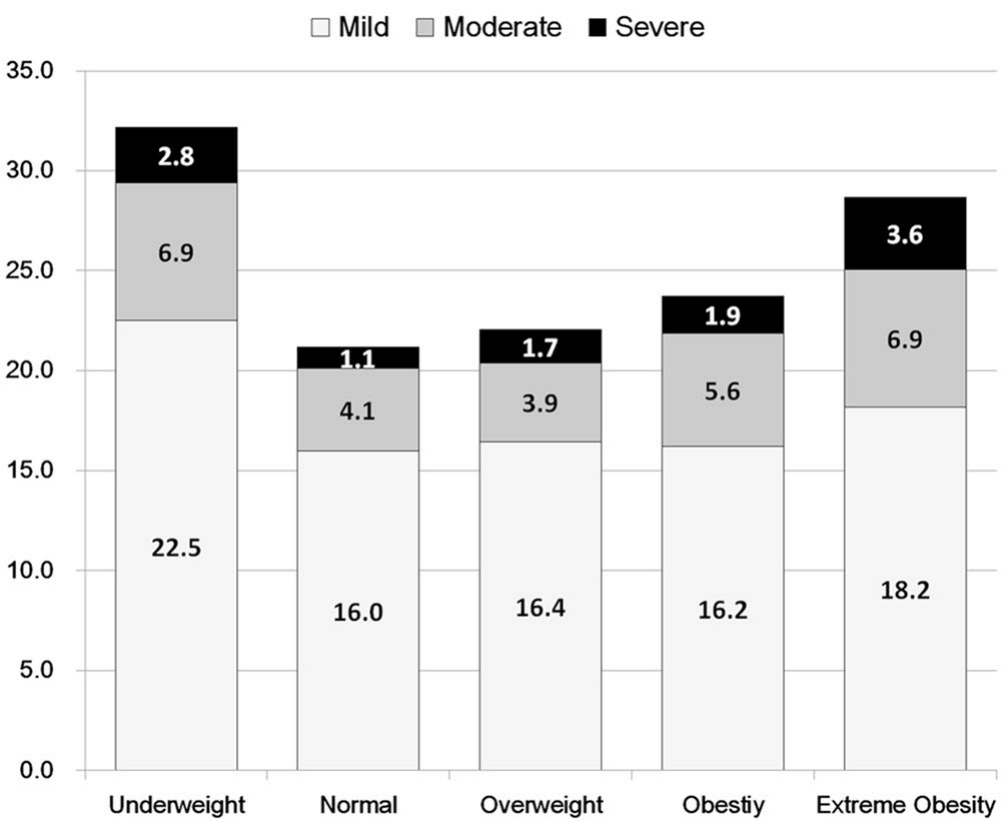
Relationships between prevalence (%) of tinnitus and BMI groups. We used the definition of the Western Pacific Region of the World Health Organization: underweight BMI < 18.5 kg/m^2^, normal weight 18.5 ≤ BMI < 23 kg/m^2^, overweight 23 ≤ BMI < 25 kg/m^2^, obese 25 ≤ BMI < 30 kg/m^2^, and severely obese BMI ≥ 30 kg/m^2^.

Overall prevalence of tinnitus was the highest in the group that was underweight, followed by the group with extreme obesity. Moderate and severe tinnitus statuses were highly prevalent in the underweight and extreme obesity groups (Fig. 2).

Table 2 lists ORs and 95% CIs for the presence of tinnitus in premenopausal women by multivariate analysis. After adjusting for all confounding variables of age, smoking, drinking, exercise, perceived stress, melancholy, and duration of sleep, women in the underweight group exhibited a higher OR for presence of tinnitus (OR, 1.54; 95% CI, 1.14–2.08) than those in the other BMI groups.

**Table 2:** Multivariate analysis of the association between tinnitus and BMI. Odds ratios (ORs) and 95% confidence intervals (CIs) for the presence of tinnitus in premenopausal women.

BMI (kg/m^2^)	Adjusted OR (95% CI)		
	Model 1	Model 2	Model 3
<18.5	1.66 (1.26–2.18)	1.50 (1.13–1.98)	1.54 (1.14–2.08)
18.5≤ and <23	Reference	reference	reference
23≤ and <25	1.02 (0.80–1.29)	1.08 (0.85–1.37)	1.05 (0.79–1.39)
25≤ and <30	1.11 (0.87–1.42)	1.18 (0.92–1.52)	1.11 (0.79–1.58)
30≤	1.33 (0.89–1.99)	1.27 (0.83–1.93)	1.08 (0.58–2.04)

Model 1 = Nonadjusted.

Model 2 = Model 1+ adjusted for age, smoking, drinking, exercise.

Model 3 = Model 2+ adjusted for perceived stress, melancholy, duration of sleep.

## Discussion

Although BMI is already known to be associated with hearing loss^8,11–13^, our findings indicated that underweight premenopausal women had an about 1.5-fold increased risk for tinnitus. This association was statistically significant even after adjusting for age, smoking, drinking, exercise, perceived stress, melancholy, and duration of sleep. This is the first study to demonstrate the relationship between tinnitus and BMI. These findings may suggest possible changes to prevent the occurrence of tinnitus and to help premenopausal women have different attitudes on their body weight.

Several studies have reported on the associations between hearing loss and BMI. Kim *et al*.^13^ evaluated the association between BMI and prevalence of hearing loss using data from 61,052 people who visited the health promotion center of a university-based hospital from 2006 to 2012. They found that underweight individuals in a Korean adult population had a higher risk for hearing loss (OR, 1.28). Lee *et al*.^12^ analyzed KNHANES data from 2010–2012 and found that larger muscle mass was associated with lower prevalence of hearing loss in older Korean females, but not Korean males. Curhan *et al*.^11^ found that lower BMI (<25 kg/m^2^) and smaller waist circumference (<71 cm) reduced the risk of hearing loss in women. Tan *et al*.^8^ demonstrated that obesity was significantly associated with hearing loss at pure-tone averages of 500, 1000, 2000, and 4000 Hz; low-frequencies of 250, 500, and 1000 Hz; and high frequencies of 4000 and 8000 Hz.

Mahboubi *et al*.^14^ analyzed NHANES data from 2005–2008 on 3520 individuals aged 12 to 19 years. They found that tinnitus was associated with female gender, low income, exposure to passive smoking, type A tympanogram, occupational and recreational noise exposure, history of ≥3 ear infections, and history of tympanostomy tube placement. In their study, BMI was not a risk factor for tinnitus. They analyzed the OR of tinnitus between BMI < 25 kg/m^2^ and BMI ≤ 25 kg/m^2^. Kim *et al*.^15^ analyzed KNHANES data from 2009–2012 on 19,290 individuals aged 20 to 98 years. They found that tinnitus was associated with gender, smoking, stress, sleep, hearing loss, hyperlipidemia, osteoarthritis, rheumatoid arthritis, asthma, depression, and thyroid disease history.

Underweight individuals may have increased chance of poor physical condition, which may lead to increased risk of morbidity and mortality. Body weight is an important factor in health status, including of the metabolic, immune, reproductive, and musculoskeletal systems. However, wrong attitudes about body weight are common in modern society. Especially, young women desire a thinner body shape and sometimes adopt excessive weight-control efforts to maintain a slim body, even if they have a normal body weight.

The reason that the prevalence of tinnitus was higher in underweight premenopausal women in this study is unclear. Kim *et al*.^13^ suggested that higher prevalence of hearing loss in underweight individuals may result from immoderate weight-reduction diets. They emphasized that nutrition imbalance could affect the pathological degeneration of the auditory system and osteogenesis, resulting in otosclerosis, imperfections in the temporal bones, decreased resistance in auditory organs, and decreased auditory recovery ability.

A recent model of tinnitus emphasizes cognitive/behavioral causes and tinnitus-related distress^16,17^. Psychoacoustic characteristics of tinnitus, such as pitch and loudness, seem to be more closely associated with psychological, emotional, and/or cognitive factors^18^. Tinnitus-related anxiety and handicap are significantly associated with mental health status, such as proneness to worry^19^, and an inverse relationship between mental health status and socioeconomic status has been demonstrated^20^. This pattern was observed in our study. In this study, the prevalence of tinnitus was higher in younger women, women who ever smoked, women in the lowest quartile of income, women living without a spouse, women with lower educational level, women without parity, and women with irregular menstruation.

This study demonstrated significant association among tinnitus, BMI, and mental health status, but we cannot explain their causal relationship in this cross-sectional study. Using KNHANES data from 2010–2012, Seo *et al*.^21^ showed that Korean patients experiencing tinnitus have higher risk of suicidal ideation and attempts. During a review of the related literature, we generated three hypotheses. The first hypothesis concerned the relationship between tinnitus and BMI: Individuals who are underweight or have extreme obesity are likely to have an unhealthy diet and resultant nutritional imbalance, which may influence the prevalence or severity of tinnitus. McCormack *et al*.^22^ reported on dietary factors involved in the presence and severity of tinnitus using a database from a large-scale cross-sectional study of 171,722 adults aged 40–69 years. Our second hypothesis was about the relationship between personality traits and BMI. Shim *et al*.^23^ reported that personality traits are associated with body weight and obesity in Koreans and found that openness to experience was negatively associated and agreeableness was positively associated with BMI in Korean women. Our last hypothesis concerned the relationship between tinnitus and personality traits. Bartle *et al*.^24^ reported a case-control study showing that tinnitus and its perceived severity are related to a type D personality trait, which refers to distressed personality that is defined by high negative affectivity and social inhibition. Adami Dehkordi *et al*.^25^ reported a case-control study showing that patients with tinnitus tend to be more neurotic and less extraverted than without tinnitus. Durai and Searchfield evaluated the association between tinnitus and personality traits such as anxiety and depression^26^. They found that anxiety and depression were more prevalent among individuals with tinnitus and concluded that personality traits were consistently associated with tinnitus-related distress. High neuroticism and lower extraversion are traditionally known to be highly associated with tinnitus perception and/or distress. Many studies report that tinnitus is associated with high-stress reactions, low social closeness, low self-control, low well-being, high alienation, high-anxiety sensitivity, low optimism, low acceptance, high external locus of control, and high type D prevalence. These studies reviewed the mechanisms by which personality traits can directly influence tinnitus-related distress. According to the signal-detection model^27^, personality traits can manipulate the response criterion placement so that individuals with increased awareness report even very low levels of signal as tinnitus. This theory is in line with the Jastreboff model^17,28^. Durai and Searchfield found that personality traits or psychiatric disorders were not likely to cause tinnitus but more likely to affect how individuals with tinnitus were distressed by amplifying symptoms and complaints^26^. Moreover, tinnitus-related distress may act as potential feedback in individuals with tinnitus. Therefore, psychiatric disorders and tinnitus may combine to form a vicious cycle and exacerbate each other.

The strengths of our study include its nationally representative data. This study analyzed data on the general population of South Korea, and our participants represent the entire South Korean population because KNHANES has rolling sampling. Additionally, the data are gathered by trained interviewers with standardized questions. Our findings are compatible with Bhatt *et al*.^2^, who analyzed a national database from the household-based 2007 National Health Interview Series. The study demonstrated that tinnitus and its severity were closely associated with anxiety and depression for the national population in the United States.

This study has several potential limitations. The first is possible recall bias because KNHANES data is gathered from self-reported questionnaires that examine respondents’ lifestyles. Second, we could not evaluate causal relations between tinnitus and BMI because our study was cross-sectional. Therefore, we invite future prospective studies on tinnitus and BMI, which will give insights about causal relationships. Third, the distinction between not-annoying and annoying tinnitus was not clear, because this study on tinnitus depended on patients’ subjective assessment. Although the survey was unbiased and guided appropriately by the interviewers, the KNHANES questions about tinnitus were simple and brief, and the degree of tinnitus might have been misclassified. Last, the prevalence of stress, melancholy, and suicide is reported to vary widely across ethnicity and socioeconomic environments^29,30^. Because this study analyzed data on South Koreans, our findings may not be applicable to other nations. Despite these weaknesses, mental health problems have been rising as an important social issue. This study may provide information representative of an entire population, with data obtained from a nationwide cohort with a high response rate.

In conclusion, underweight premenopausal women were at higher risk of tinnitus, which is a major public health issue for younger through older people. Because underweight women with tinnitus experience frequent perceived stress and suicide ideation, our study also emphasizes the need for attention to their health. Underweight may represent a surrogate marker for tinnitus in premenopausal women. Our findings support promoting normal body weight and maintaining normal BMI as helpful for relieving tinnitus.

## Electronic supplementary material

Supplementary Table S1.

## References

[1] Bhatt JM, Lin HW, Bhattacharyya N (2016). Prevalence, Severity, Exposures, and Treatment Patterns of Tinnitus in the United States. JAMA Otolaryngol Head Neck Surg.

[2] Bhatt JM, Bhattacharyya N, Lin HW (2017). Relationships Between Tinnitus and the Prevalence of Anxiety and Depression. Laryngoscope.

[3] Park KH (2014). Prevalence and associated factors of tinnitus: data from the Korean National Health and Nutrition Examination Survey 2009-2011. J Epidemiol.

[4] Nondahl DM (2010). The ten-year incidence of tinnitus among older adults. Int J Audiol.

[5] Park B (2014). Analysis of the prevalence of and risk factors for tinnitus in a young population. Otol Neurotol.

[6] Gallus S (2015). Prevalence and Determinants of Tinnitus in the Italian Adult Population. Neuroepidemiology.

[7] Martines F (2015). Clinical observations and risk factors for tinnitus in a Sicilian cohort. Eur Arch Otorhinolaryngol.

[8] Tan HE (2018). Associations between cardiovascular disease and its risk factors with hearing loss-A cross-sectional analysis. Clin Otolaryngol.

[9] Lee SS, Han KD, Joo YH (2017). Association of perceived tinnitus with duration of hormone replacement therapy in Korean postmenopausal women: a cross-sectional study. BMJ Open.

[10] Regional Office for the Western Pacific, W. *The Asia Pacific perspective: Redefining obesity and its treatment*. (Sydney: Health Communications Australia, 2000).

[11] Curhan SG, Eavey R, Wang M, Stampfer MJ, Curhan GC (2013). Body mass index, waist circumference, physical activity, and risk of hearing loss in women. Am J Med.

[12] Lee J, Han K, Song JJ, Im GJ, Chae SW (2016). Sarcopenia and Hearing Loss in Older Koreans: Findings from the Korea National Health and Nutrition Examination Survey (KNHANES) 2010. PLoS One.

[13] Kim SH (2016). Relationship between obesity and hearing loss. Acta Otolaryngol.

[14] Mahboubi H, Oliaei S, Kiumehr S, Dwabe S, Djalilian HR (2013). The prevalence and characteristics of tinnitus in the youth population of the United States. Laryngoscope.

[15] Kim HJ (2015). Analysis of the prevalence and associated risk factors of tinnitus in adults. PLoS One.

[16] Hallam, R. S., Rachman, S. & Hinchcliffe, R. In *Contributions to medical psychology* (ed S. Rachman) 31–53 (Pergamon Press, 1984).

[17] Jastreboff PJ (1990). Phantom auditory perception (tinnitus): mechanisms of generation and perception. Neurosci Res.

[18] Balkenhol T, Wallhausser-Franke E, Delb W (2013). Psychoacoustic tinnitus loudness and tinnitus-related distress show different associations with oscillatory brain activity. PLoS One.

[19] Caldirola D (2016). Role of worry in patients with chronic tinnitus and sensorineural hearing loss: a preliminary study. Eur Arch Otorhinolaryngol.

[20] Hudson CG (2005). Socioeconomic status and mental illness: tests of the social causation and selection hypotheses. Am J Orthopsychiatry.

[21] Seo JH, Kang JM, Hwang SH, Han KD, Joo YH (2016). Relationship between tinnitus and suicidal behaviour in Korean men and women: a cross-sectional study. Clin Otolaryngol.

[22] McCormack A (2014). Association of dietary factors with presence and severity of tinnitus in a middle-aged UK population. PLoS One.

[23] Shim U (2014). Personality traits and body mass index in a Korean population. PLoS One.

[24] Bartels H, Middel B, Pedersen SS, Staal MJ, Albers FWJ (2010). The Distressed (Type D) Personality Is Independently Associated With Tinnitus: A Case-Control Study. Psychosomatics.

[25] Adami Dehkordi M, Javanbakht M, Sarfarazi Moghadam S, Meshkat M, Abolbashari S (2015). Personality Traits in Patients with Subjective Idiopathic Tinnitus. Iran J Otorhinolaryngol.

[26] Durai M, Searchfield G (2016). Anxiety and depression, personality traits relevant to tinnitus: A scoping review. International journal of audiology.

[27] Welch D, Dawes PJ (2008). Personality and perception of tinnitus. Ear Hear.

[28] Ghodratitoostani I (2016). Theoretical Tinnitus Framework: A Neurofunctional Model. Front Neurosci.

[29] Health: Key Tables from OECD. 17. Suicides Deaths per 100,000 population. http://dx.doi.org/10.1787/20758480-table 10 [accessed on 30 June 2014] (2014).

[30] Health status: Suicide rates. http://dx.doi.org/10.1787/bd12d298-en [accessed on 10 Nov 2017] (2017).

