# Supplementary material for: Nationwide analysis of the relationships between mental health, body mass index and tinnitus in premenopausal female adults in Korea: 2010–2012 KNHANES
Source: Sci Rep. 2018 May 4;8:7028. doi: 10.1038/s41598-018-25576-5 (PMC5935674; doi:10.1038/s41598-018-25576-5)
Supplement: Supplementary file 1 — Supplementary Table S1. [file 41598_2018_25576_MOESM1_ESM.docx]

**SUPPLEMENTARY INFORMATION**

**Nationwide analysis of the relationship between body mass index and tinnitus in premenopausal female adults in Korea: 2010–2012 KNHANES**

Dong-Hee Lee,^1,2^ Young Soo Kim,^1,3^ Hiun Suk Chae,^1,3^ and Kyungdo Han^4^

^1^ Epidemiology Study Cluster of Uijeongbu St. Mary's Hospital, Uijeongbu St. Mary’s Hospital, College of Medicine, The Catholic University of Korea, Uijeongbu, Korea

^2^ Department of Otolaryngology-HNS, College of Medicine, The Catholic University of Korea, Seoul, Korea

^3^ Department of Internal medicine, College of Medicine, The Catholic University of Korea, Seoul, Korea

^4^ Department of Biostatistics, College of Medicine, The Catholic University of Korea, Seoul, Korea

Corresponding Author: **Dong-Hee Lee**

Department of Otolaryngology-Head and Neck Surgery, Uijeongbu St. Mary’s Hospital, College of Medicine, The Catholic University of Korea, 271 Cheonbo Street, Uijeongbu city, Gyeonggi-do, 11765, Republic of Korea

Tel: 82-31-820-3564, Fax: 82-31-847-0038

E-mail: leedh0814@catholic.ac.kr

**Supplementary Table S1.** (A) Summary of 3 mental health dimensions in the study population. Prevalence of stress, melancholy, and suicidal ideation (B) according to tinnitus or BMI and (C) according to tinnitus and underweight. Data are presented as % (SE).

(A)

| BMI (kg/m^2^) | Perceived stress | | Melancholy | | Suicide ideation | |
| --- | --- | --- | --- | --- | --- | --- |
|  | No | Yes | No | Yes | No | Yes |
| <18.5 | N=271 | N=160 | N=374 | N=57 | N=361 | N=70 |
| 18.5≤ and <23 | N=1686 | N=759 | N=2110 | N=335 | N=2102 | N=343 |
| 23≤ and <25 | N=541 | N=241 | N=678 | N=104 | N=677 | N=105 |
| 25≤ and <30 | N=530 | N=253 | N=672 | N=111 | N=674 | N=108 |
| 30≤ | N=108 | N=79 | N=153 | N=34 | N=141 | N=46 |
| Total | N=3136 | N=1492 | N=3987 | N=641 | N=3955 | N=672 |

(B)

|  | Tinnitus | | |  | BMI (kg/m^2^) | | | | | |
| --- | --- | --- | --- | --- | --- | --- | --- | --- | --- | --- |
|  | No | Yes | P-value |  | <18.5 | 18.5≤  <23 | 23≤  <25 | 25≤  <30 | 30≤ | P-value |
| Perceived stress | 31.3  (1.0) | 41.8  (2.0) | <0.001 |  | 38.5  (2.8) | 32.2  (1.1) | 31.5  (1.9) | 33.7  (2.1) | 45.1  (4.3) | 0.007 |
| Melancholy | 13.0  (0.6) | 20.0  (1.6) | <0.001 |  | 12.6  (1.8) | 14.4  (0.8) | 14.4  (1.4) | 14.6  (1.6) | 20.8  (3.5) | 0.231 |
| Suicide ideation | 13.6  (0.7) | 24.3  (1.8) | <0.001 |  | 16.3  (2.1) | 15.4  (0.9) | 15.0  (1.6) | 14.5  (1.4) | 29.8  (4.0) | <0.001 |

(C)

| Underweight | No | | Yes | | P-value |
| --- | --- | --- | --- | --- | --- |
| Tinnitus | No | Yes | No | Yes |  |
| Perceived stress | 30.9(1.0) | 40.9(2.1) | 34.7(3.4) | 47.6(5.6) | <0.001 |
| Melancholy | 13.0(0.7) | 21.4(1.8) | 13.1(2.2) | 11.3(3.3) | <0.001 |
| Suicide ideation | 13.5(0.7) | 24.8(1.8) | 14.4(2.3) | 20.9(4.7) | <0.001 |
